# 587. An Intervention to Improve COVID-19 Vaccination Rates Among Inpatients at a Veterans Affairs Hospital

**DOI:** 10.1093/ofid/ofab466.785

**Published:** 2021-12-04

**Authors:** Ayako Fujita, Tiffany Goolsby, Krista Powell, Emily J Cartwright

**Affiliations:** 1 Emory University School of Medicine, Atlanta, Georgia; 2 Atlanta VA Health Care System, Atlanta, Georgia; 3 Atlanta VA Medical Center, Atlanta, Georgia

## Abstract

**Background:**

Hospitalizations are an opportunity to increase vaccine uptake and hospital-based strategies have been effective at increasing influenza and pneumococcal vaccination. Offering COVID-19 vaccination at discharge can reduce barriers to vaccination and target patients at high risk for severe illness and death. We evaluated a COVID-19 vaccine intervention implemented as part of routine discharge planning.

**Methods:**

We trained healthcare personnel during April 2021 to review and document vaccine eligibility and interest for adult inpatients on medical, surgical, or psychiatric wards at the Atlanta VA Medical Center during discharge planning using a templated note in the electronic medical record (EMR). Outpatient vaccination center personnel were deployed to the participating wards daily (except Sundays) to facilitate vaccine administration at discharge. We measured the percentage of discharged patients with vaccine eligibility documented using the template and compared the number of patients vaccinated at discharge in the 4 weeks pre- and post-training. All Georgia adults became eligible for COVID-19 vaccines on March 25, 2021, prior to our intervention.

**Results:**

Of the 769 patients discharged from one of the participating wards during the 4-week post-training, 474 (62%) had vaccine eligibility documented (Table 1). Of the 474 patients with documentation, 88 (19%) were eligible. Reasons for ineligibility included prior vaccination (n=266, 69%), patient refusal (n=103, 27%), and acute COVID infection (n=12, 3%). Of the 88 eligible patients, 61 (69%) received vaccination before discharge. In total, 16 of 793 inpatients in the pre-training period and 61 of 769 in the post-training period (2% vs 8%; p< 0.05) were vaccinated prior to discharge.

Table 1. COVID-19 vaccine eligibility and vaccination before discharge during the post-training period, reported by week

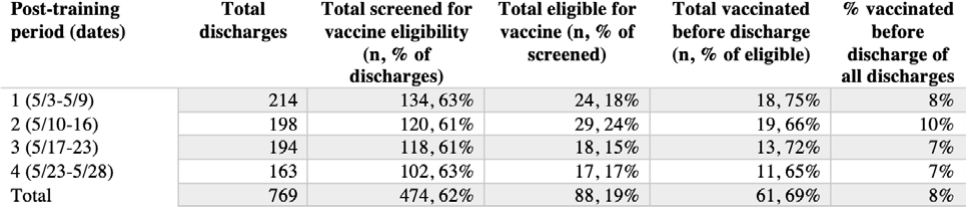

**Conclusion:**

We found relatively high and sustained uptake of an intervention to screen hospitalized patients for COVID-19 vaccination eligibility. Creating a templated note in the EMR resulted in vaccination of nearly 70% of eligible patients prior to hospital discharge.

**Disclosures:**

**All Authors**: No reported disclosures

